# European Headache Federation recommendations for neurologists managing giant cell arteritis

**DOI:** 10.1186/s10194-020-01093-7

**Published:** 2020-03-17

**Authors:** S. P. Mollan, K. Paemeleire, J. Versijpt, R. Luqmani, A. J. Sinclair

**Affiliations:** 1 Birmingham Neuro-Ophthalmology, University Hospitals Birmingham NHS Foundation Trust, Queen Elizabeth Hospital, Birmingham, UK; 2 Department of Neurology, Ghent University Hospital, Ghent, Belgium; 3 Department of Neurology, Vrije Universiteit Brussel (VUB), Universitair Ziekenhuis Brussel (UZ Brussel), Brussels, Belgium; 4 The Nuffield Department of Orthopaedics, Rheumatology and Musculoskeletal Sciences, Kennedy Institute of Rheumatology, Roosevelt Drive, Headington, Oxford, OX3 7FY UK; 5 Metabolic Neurology, Institute of Metabolism and Systems Research, University of Birmingham, Edgbaston, Birmingham, B15 2TT UK; 6 Department of Neurology, University Hospitals Birmingham, Queen Elizabeth Hospital, Birmingham, UK; 7 Centre for Endocrinology, Diabetes and Metabolism, Birmingham Health Partners, Birmingham, UK

**Keywords:** Giant cell arteritis, Temporal arteritis, Headache, Large vessel Vasculitis, Polymyalgia Rheumatica, Tocilizumab, Vision, Anterior Ischaemic optic neuropathy, Stroke

## Abstract

**Background and aim:**

Giant cell arteritis (GCA) remains a medical emergency because of the risk of sudden irreversible sight loss and rarely stroke along with other complications. Because headache is one of the cardinal symptoms of cranial GCA, neurologists need to be up to date with the advances in investigation and management of this condition. The aim of this document by the European Headache Federation (EHF) is to provide an evidence-based and expert-based recommendations on GCA.

**Methods:**

The working group identified relevant questions, performed systematic literature review and assessed the quality of available evidence, and wrote recommendations. Where there was not a high level of evidence, the multidisciplinary (neurology, ophthalmology and rheumatology) group recommended best practice based on their clinical experience.

**Results:**

Across Europe, fast track pathways and the utility of advanced imaging techniques are helping to reduce diagnostic delay and uncertainty, with improved clinical outcomes for patients. GCA is treated with high dose glucocorticoids (GC) as a first line agent however long-term GC toxicity is one of the key concerns for clinicians and patients. The first phase 2 and phase 3 randomised controlled trials of Tocilizumab, an IL-6 receptor antagonist, have been published. It is now been approved as the first ever licensed drug to be used in GCA.

**Conclusion:**

The present article will outline recent advances made in the diagnosis and management of GCA.

## Objective

To systematically review the literature for advances in the diagnosis and management of Giant cell arteritis (GCA), in order to provide practical guidance statements for the neurologist (which concords with and complements guidelines from other specialties).

## Methods

The European Headache Federation (EHF) board identified GCA as a disease area where new evidence has emerged. The working group was put together to include neurologists with a specialist interest in headache and an ophthalmologist. The group identified relevant questions then performed a systematic literature review. The literature search included all English papers on PubMed between inception of the database until July 1st, 2019, a further search was performed on January 17th, 2020 to ensure all relevant papers could be included. The papers were assessed for their quality and the recommendations drafted. The draft recommendations were critically reviewed by a rheumatologist, who became part of the EHF GCA panel. The final document was reviewed and approved by all members of the panel. Where there was not a high level of evidence, the multidisciplinary EHF GCA panel recommended best practice based on their clinical experience and that of other specialty guideline groups.

## Background

GCA is the cause of a critical secondary headache, that if left undiagnosed has serious permanent consequences for the patient [1]. It is the commonest form of systemic granulomatous vasculitis [2] and the immunopathophysiology is well described [3, 4]. It is likely that both genetics and environmental factors are important in initiating the inflammatory cascade [5, 6].

The incidence of GCA is between 15 to 25 cases per 100,000 persons over 50 years of age, and increases with age [7]. It more commonly affects women with a lifetime risk of GCA in women of 1% compared to 0.5% in men [8]. It is a disease of Caucasians and has a higher incidence in Scandinavian countries and in populations of Northern European descent [9].

**Fig. 1:**
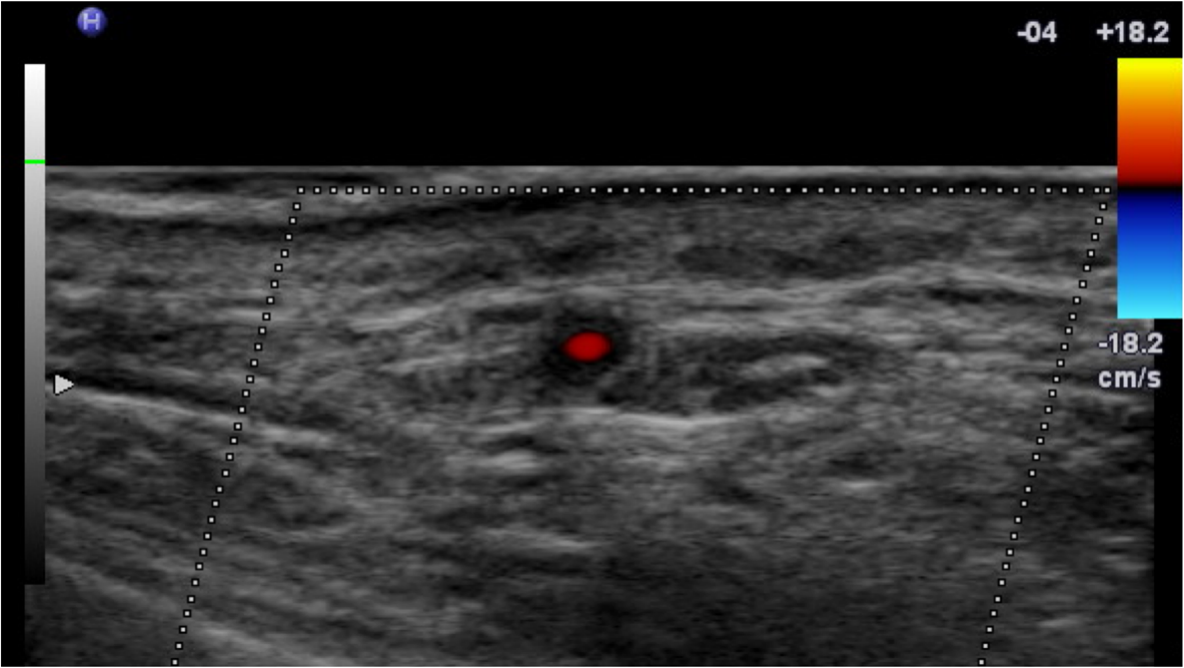
CDUS of the right temporal artery with a hyporeflective « halo » around the temporal artery in a patient with subsequently a positive temporal artery biopsy

Due to the risk of sudden permanent sight loss in between 8 and 30% [10, 11], and stroke in between 3 and 10% [12], GCA is a medical emergency. GCA is classified as a large vessel vasculitis (LVV) as defined by the Chapel Hill Consensus Definitions because it affects the aorta and its major branches; however, any size artery may be affected, such as small ocular and periocular arteries that lead to visual loss [2]. It is now known to be a spectrum of phenotypically overlapping conditions including cranial GCA (previously known as temporal arteritis), and extra-cranial GCA otherwise termed large vessel-GCA (LV-GCA), usually involving the aorta and its larger supra-aortic branches, and polymyalgia rheumatica (PMR) [13]. However, any individual patient may have an overlap of more than one subtype (case vignette 1, Fig. 1). The most commonly affected cranial arteries are the temporal, ophthalmic, and posterior ciliary arteries causing visual loss, and rarely the vertebral or carotid territories that can lead to stroke [14].

Clinically it can be difficult to diagnose, because the symptoms can be insidious [15, 16]. Evidence suggests that increasing numbers of patients are being investigated for suspected GCA [17]. Across Europe, rheumatology centres have set up rapid access investigation pathways, resulting in improved outcomes for patients [18]. Advancing imaging techniques have demonstrated a larger portion of patients with large vessel involvement both with auxiliary ultrasound [19] and formal large vessel imaging studies [20]. In management of the disease, we now acknowledge that many patients with GCA are exposed to high cumulative doses of glucocorticoids (GC), resulting in significant increasing long-term [21]. The first ever therapy, Tocilizumab, that is specifically licensed for GCA and may be used as a GC-sparing agent, was given regulatory approval in 2017. GCA is moving from a condition managed by many specialists to a disease requiring expertise in both the diagnosis and long-term management of the condition.

This timely guidance for neurologists is based on the formation of questions to allow systematic interrogation of the current literature. The two main themes include diagnosis and treatment of GCA.

## What are the characteristics of headache in GCA?

The International Headache Society (IHS) definition of headache attributable to GCA requires two of the following to be fulfilled in any new headache:

1. headache has developed in temporal relation to other symptoms and/or clinical or biological signs of onset of GCA, or has led to the diagnosis of GCA
2. either or both of the following:
  ▪ a) headache has significantly worsened in parallel with worsening of GCA
  ▪ b) headache has significantly improved or resolved within 3 days of high-dose steroid treatment
3. headache is associated with scalp tenderness and/or jaw claudication [22].

The IHS comment that the variability in the headache and other symptoms of GCA are such that any recent persisting headache in a patient over 60 years of age should suggest GCA and lead to appropriate investigations [22]. The IHS classification criteria are informed by the literature, and will likely evolve as more is known about headache in GCA. Caution should be applied not to use criterion 2b in isolation, for example many different types of headaches can improve with initiation of high dose GC treatment.

New onset headache is a cardinal symptom of GCA, with 67% reporting this symptom in the largest trial of GCA to date [23]. Headache alone may be the initial symptom for those with cranial GCA, and as the undiagnosed disease progresses, it becomes a more commonly reported symptom [24]. The full headache phenotype is little further described than “new onset” in the majority of the literature, and therefore when used in isolation, it appears to be only a modest discriminator when trying to predict the likelihood of GCA [25].

In a study from Japan, the headache has been reported as continuous in 60% of patients, with just under half having paroxysmal headache [26]. Case reports suggest that the headache is severe and unlike prior headaches in those who have had a prior history of headache [27]. However, there is a spectrum of severity of the pain, and the Japanese series has reported a range from severe (42%), to moderate (37%) and mild (21%) headache [26].

The location of pain is commonly reported as being in the temporal artery (TA) region, when the TA is involved and may be more holocranial in nature in others, which probably reflects the arterial involvement of the disease [28]. Nineteen cases of GCA that were reviewed at the Japanese headache centre reported the location of the headache to be 60% temporal, 11% occipital, 11% frontal, 5% generalised. Of the remaining 15% no headache was recorded, one patient had isolated ear pain, and the other isolated jaw pain [26]. Importantly headache is also a common symptom at relapse [29].

### Recommendation

The GCA headache phenotype is yet to be fully characterized in terms of frequency, severity and other associated characteristics. Vigilance is warranted in patients with new onset headache over the age of 50 years.

## What are the presenting features of GCA, apart from new onset headache?

There is major overlap of symptoms between the three sub-types of GCA, namely: cranial GCA, LV-GCA and PMR. Cranial signs and symptoms include temporal cutaneous hyperalgesia, jaw or tongue claudication, abnormalities of the temporal artery on examination (prominent, beaded or irregular artery with a decreased pulse in up to one third of patients), scalp or tongue necrosis, tongue necrosis, and rarely stroke. Up to 30% develop ophthalmic signs and symptoms including transient monocular visual loss (amaurosis fugax) up to permanent loss of vision due to:

- Anterior ischaemic optic neuropathy
- Central retinal artery occlusion
- Branch retinal artery occlusion
- Posterior ischaemic optic neuropathy
- Choroidal infarction

Other visual symptoms include transient diplopia and persistent diplopia, secondary to extra-ocular muscle ischemia, isolated or multiple oculomotor cranial nerve palsies [4]. Systemic symptoms include fever, myalgia, fatigue, night sweats, loss of appetite, unintentional weight loss, and mood change [4]. Large vessel manifestations include aortitis, limb claudication (due to stenosis), thoracic and abdominal aneurysms.

### Recommendation

A comprehensive history and examination in all those suspected with GCA will help guide investigations.

## What is the best way to diagnose GCA?

Diagnosing GCA can be challenging and requires a full history for clinical features, thorough examination and a combination of investigations. There are classification criteria, but no universally accepted diagnostic criteria [30]. Choosing the optimal investigations depends on which area of the body the disease is suspected to be present because one third of LV-GCA patients have no ultrasonographic evidence of cranial/temporal arteritis [19], and more than half of LV-GCA patients have a normal temporal artery biopsy [31].

### Laboratory markers

There is currently no specific blood biomarker that can definitely diagnose GCA. The majority of those with GCA have elevated acute phase reactants at disease onset such as an elevated erythrocyte sedimentation rate (ESR) [32] or plasma viscosity, C-reactive protein (CRP) [32] and platelets [33]. However inflammatory markers have been reported as normal in between 1 and 10% [32, 34]. Additionally, few cases who have presented with visual loss within the literature have reported normal inflammatory markers [35, 36]. It is well known that those with cranial GCA typically have lower inflammatory markers than patients with extracranial manifestations (LV-GCA and PMR) [24, 37, 38]. Additionally, those with higher inflammatory markers tend to have fewer ischaemic events [39]. Other blood changes include anaemia and elevated liver enzymes.

#### Recommendation

A combination of full blood count, ESR (or plasma viscosity) and CRP should be performed in those with suspected GCA which may support the clinical diagnosis.

### Confirmatory investigational tests

Both standard treatment with GC [40] and indeed licensed targeted treatment [41] for GCA confers significant risk of morbidity, therefore a confirmatory investigational test should be performed to secure the diagnosis.

#### Recommendation

A confirmatory investigational test should be performed where possible in all those with suspected GCA.

### Imaging – cranial GCA

Colour Duplex Ultrasonography (CDUS) is a non-invasive imaging modality that assesses the arterial wall anatomy, the patency of the lumen and allows assessment of blood flow. In GCA, imaging the temporal arteries shows homogenous, hypoechogenic (dark), circumferential vessel wall thickening, which is known as the “halo sign” when pictured in cross-section (Fig. 1) [42]. On meta-analysis the sensitivity and specificity of a unilateral hypoechoic halo compared to positive TAB were respectively of 68% (95% Confidence Intervals (CI): 57–78) and 81%. The diagnostic value of any abnormality on CDUS, such as hypoechoic halo and/or stenosis and/or occlusion, compared to TAB had a sensitivity of 78% (95% CI, 64–87), a specificity of 79% [43].

Other features on examination include the compression sign when using b-scan mode, where a normal artery image is extinguished on compression whilst an artery with features of active vasculitis is not, providing superior inter-observer agreement [44]. This is important in distinguishing patients with artefactual changes which lead to the detection of false halos (such as the presence of extensive vessel tortuosity and/or atherosclerosis) from a true halo.

Major advantages of CDUS are that it is non-invasive and can sample the majority of main arterial territories which can be affected such as the common superficial temporal artery, frontal and parietal branches as well as axillary, carotid, subclavian and vertebral arteries [45, 46]. The Temporal Artery Biopsy vs Ultrasound in Diagnosis of Giant Cell Arteritis (TABUL) study, a cross-sectional prospective study of 381 patients investigated for GCA, has given the most comprehensive analysis of use of CDUS in GCA [45]; with this evidence CDUS has allowed for a significant reduction in the requirement for temporal artery biopsy [47].

**Fig. 2:**
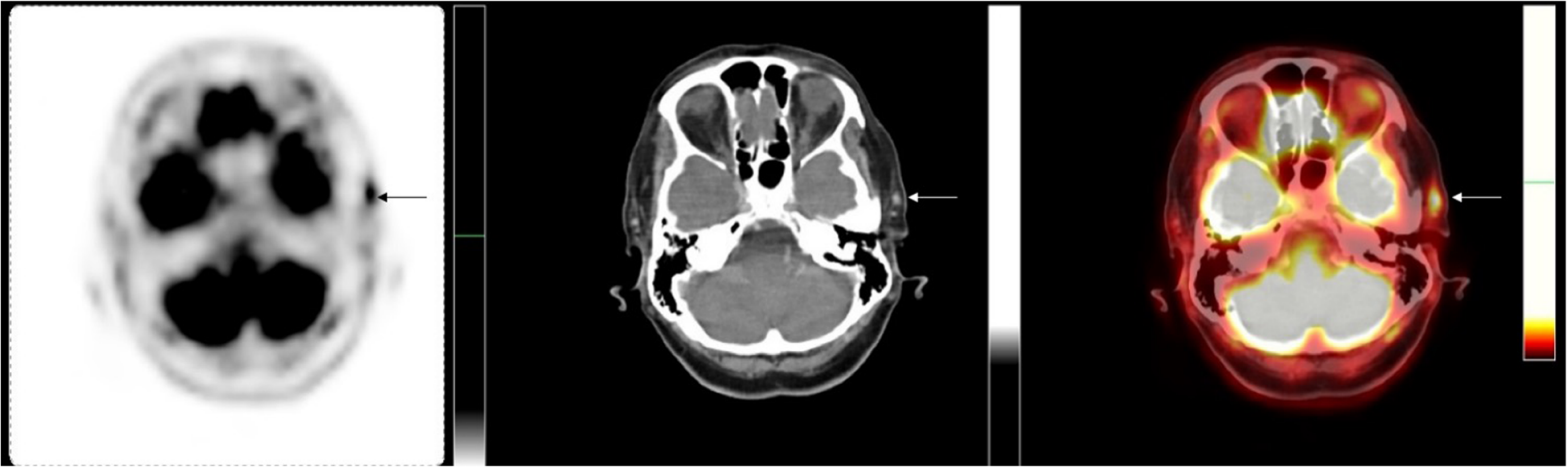
Increased FDG uptake in the temporal arteries, more pronounced on the left. Images from left to right: FDG-PET, CT (soft window), and fusion

^18^F-fluorodeoxyglucose positron emission tomography (^18^F-FDG-PET) has traditionally been technically difficult to interpret in cranial disease due to the brain activity and the size of the temporal vessels being assessed. Recently the GAP study [48], reported on a progressive protocol to assess the head, neck and thorax with a dedicated time-of-flight 18F-FDG-PET protocol and 1 mm CT reconstruction. They found that it was helpful early in the investigational pathway not only to distinguish masquerades of GCA but also for distinguishing active disease in the intracranial vessels (Fig. 2). However, major disadvantages of this modality remain such as the cost, radiation exposure, availability and validation with existing imaging modalities.

#### Recommendation

Where CDUS is readily available, it should be used to confirm the clinical diagnosis of GCA.

### Temporal artery biopsy

Temporal artery biopsy (TAB) allows for a histological diagnosis, but in some centres may be difficult to access readily and for the patient, it is an invasive procedure. When a TAB is positive it provides justification for treatments used in GCA, however when negative can create diagnostic uncertainty. The sensitivity of TAB ranges in the literature between 80 and 90% probably due to publication bias, however in a comparative trial, it was shown to have a poor sensitivity of 39% [45]. Negative biopsies can be a concern for the clinician, and a number of factors may contribute including the nature of the tissue obtained (i.e vein instead of artery biopsied), length of biopsy (preferably greater than 1 cm), histological interpretation and length of time on GC treatment resulting in a false negative biopsy [45, 49]. However, given the morbidity of treatment, some would still consider performing a biopsy at any point during the disease [50]. The utility of TAB has been recommended by some international bodies to be superseded by non-invasive imaging modalities such as CDUS [20]. Some centres have chosen to use TAB when the results of CDUS are either equivocal or not in keeping with the clinical presentation [51].

#### Recommendation

Temporal artery biopsy remains a useful and specific investigation for a definitive diagnosis of GCA.

### Imaging – large vessel GCA (LV-GCA)

LV-GCA often presents as an inflammatory syndrome and is only detected by imaging modalities such as: CDUS, computed tomography (CT) / CT angiography (CTA), magnetic resonance imaging/angiography (MRI/MRA) or ^18^F-FDG-PET. CDUS shows axillary and carotid artery circumferential homogenous hypoechogenic wall thickening, which is similar to the findings in the temporal arteries when affected [19]. However, CDUS lacks the ability to image the larger thoracic vessels.

**Fig. 3:**
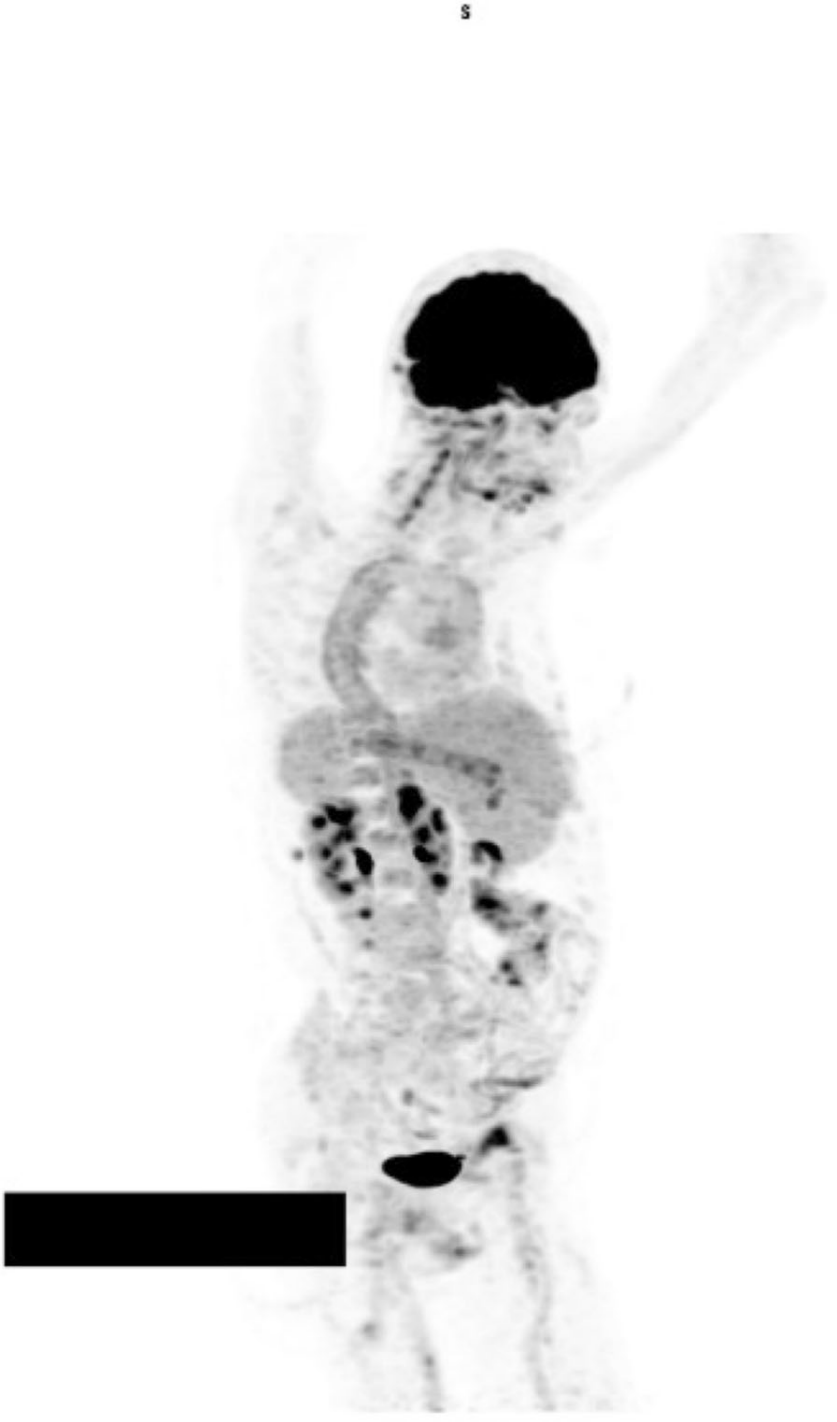
Whole body FDG-PET image illustrating increased metabolic activity in the aorta (most pronounced in the descending part) as well as some of its main branches (including both vertebral arteries, more pronounced on the left)

**Fig. 4:**
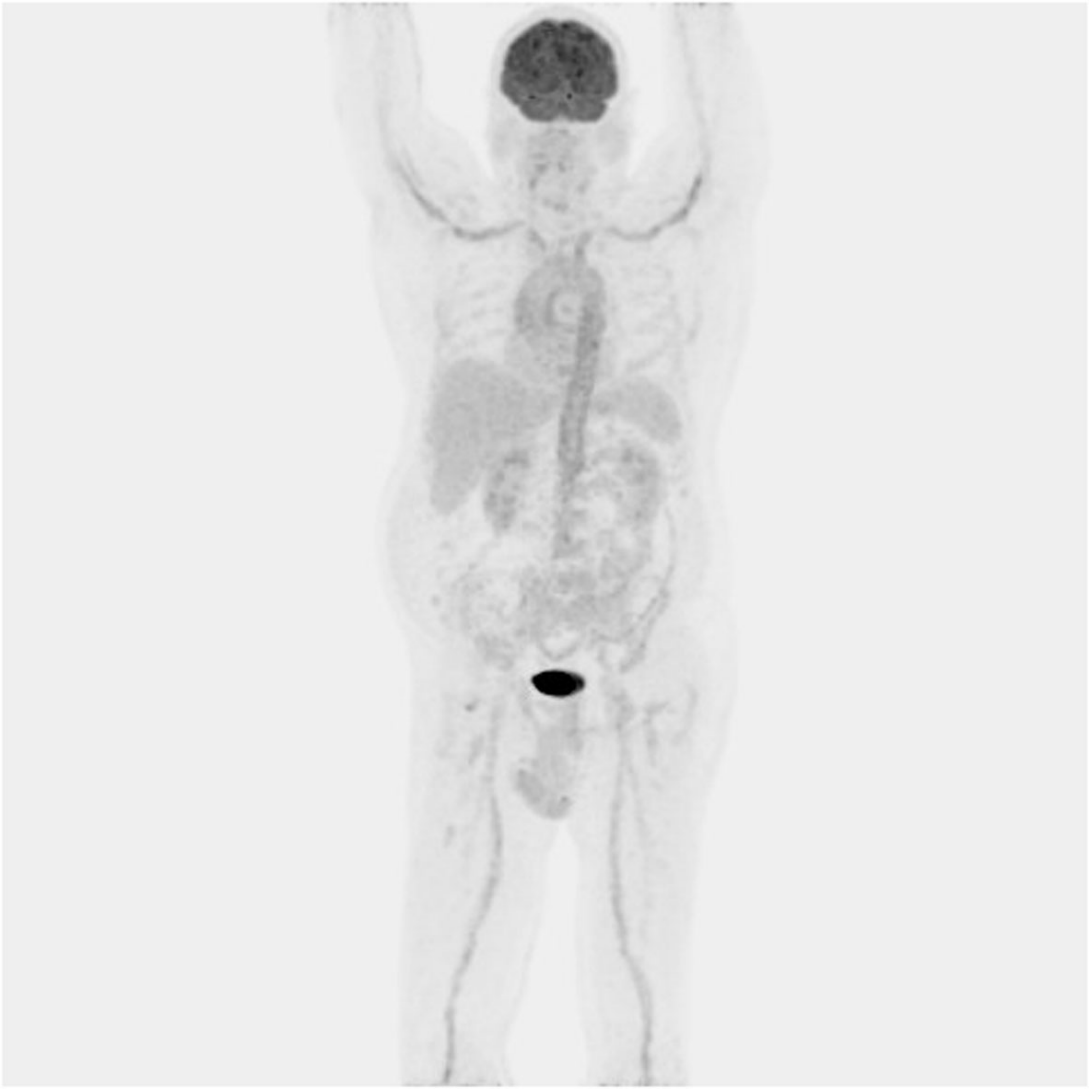
Whole body FDG-PET image illustrating increased metabolic activity in the aorta wall as well as some of its main branches including both axillary arteries

^18^F-FDG-PET is usually combined with low-dose CT and has a role in assessing disease activity and the extent of involvement in extracranial GCA (case vignette 1 and 2, Figs. 2, 3 and 4) [52]. GCA with a reported sensitivity of 77% [53]. Increased FDG uptake in the vessel wall is the hall-mark of vasculitis in PET. In general, visual evidence of vascular uptake higher than tracer uptake in the liver is considered to be suspicious for LVV [54]. ^18^F-FDG-PET imaging can demonstrate involvement of the larger aortic vessels and increased reliability. Moreover, the increased uptake seems to persist longer after treatment initiation.

#### Recommendation

Where extra-cranial disease is suspected ^18^F-FDG-PET/CT should be performed.

### Are there any other imaging modalities being utilised in GCA?

High-resolution (3 up to 7 Tesla) MRI to image the superficial and extracranial arteries in GCA demonstrated arterial wall thickening with peri-adventitial and mural contrast enhancement with sensitivities ranging from 50 to 89% [55]. Other imaging techniques that are being investigated include photoacoustic imaging [56] and optical coherence tomography of the superficial temporal artery [57].

## What are the benefits of rapid access GCA diagnostic pathways?

The mean diagnostic delay for those with cranial symptoms is 9 weeks and for those presenting without cranial symptoms is over 17 weeks. There are many factors leading to delay, including delayed presentation, low clinical suspicion, belated referral for specialist assessment, and lack of access to confirmatory diagnostic tests [15]. General public awareness of GCA is low and the early symptoms of GCA are non-specific. If GC are started, but there is a delay in performing a confirmatory investigational test then the potential for a secure diagnosis is reduced, because both CDUS and TAB are less likely to be positive with increasing time on high dose glucocorticoid therapy. The accuracy of biopsy was likely to be greatest if performed within 3 days of starting steroids (sensitivity 48% vs 33% for biopsies performed 7 or more days after commencing steroid treatment). For ultrasound, the accuracy was highest for patients seen on no more than one dose of steroids (sensitivity 64%), but was still maintained at 47% up to 7 days after commencing high dose GC [45]. The literature shows improved patient outcomes for those who have rapid access pathways [18, 58] what is yet to be demonstrated is the economic benefit of these specialists centres.

### Recommendation

- To reduce diagnostic delay, improve likelihood for securing diagnosis and improve patient outcomes, patients with suspected GCA should be referred to a rapid access specialist GCA service where available.
- Where ophthalmic symptoms are present, emergency referral to an ophthalmologist should be sought to confirm the nature of the visual symptoms.

## What is the treatment of GCA?

### What is the immediate treatment for GCA?

Where there is a high clinical suspicion of GCA immediate high dose GC should be started [59]. The standard initial GC dose for GCA is between 40 and 60 mg oral prednisolone equivalent per day, depending on the patient characteristics, including pre-existing comorbidities and body weight.

For those with cranial ischaemic symptoms (such as visual loss secondary to GCA or stroke) high dose intravenous methylprednisolone 500 mg–1000 mg induction therapy for 3 days may be used, followed by reducing course of oral glucocorticoids.

The little evidence in the literature to guide the route of administration of glucocorticoids is conflicting. Mazlumzadeh et al [60] found that a 3-day induction of IV methylprednisolone allowed a more rapid weaning from oral prednisone than placebo and conferred a reduced the cumulative glucocorticoid dose at Week 78. However, Chevalet et al [61] showed no benefit for a single induction dose of IV methylprednisolone in reducing cumulative steroid dose at 1 year. Hayreh et al. [62] in his observational case series did not show any obvious benefit between IV or oral administration, although those treated with IV tended to have poorer visual acuity at diagnosis.

Worryingly, there is evidence that sight loss can still occur in nearly one third, despite the use of high dose IV methylprednisolone within the first 6 days [63].

## Recommendation

High dose GC should be started immediately for those with a high index of clinical suspicion for GCA.

### What is the long-term GCA treatment?

Data from the GiACTA trial [64] reported that only 14% of patients on a short 6 month oral GC taper (without additional tocilizumab) achieved remission at 12 months, and 18% achieved remission at 12 months, if given a 12 month GC tapering regimen. The EULAR guidelines have recommended maintaining induction levels of 40-60 mg per day until there is clear resolution of symptoms and normalization of inflammatory markers. GC should be tapered to a target of 15–20 mg/day within 2–3 months and then to ≤5 mg/day after 1 year, with an aim to stop within 1–2 years if there is no relapse; this strategy would result in a projected cumulative dose of just over 6 g [59].

## Recommendation

GC are used for 6–24 months and should be tapered according to response.

### How are the side-effects of glucocorticoids mitigated?

The prolonged use of GC is well documented and can exacerbate conditions such as hypertension, diabetes mellitus, heart failure, and osteoporosis. It can also cause depression [65]. In patients with GCA there is a high morbidity with 86% developing at least one side effect and 68% developing more than two [40]. Real world cumulative dose of GC can far exceed guideline recommendations, and one study showed a mean cumulative dose of 8.4 g, with more than one third accumulating a dose greater than 10 g [66]. This is likely because disease relapses are common, reported to occur in half of all patients, necessitating an increase in GC, or a delay in tapering the dose [67]. For those with a history of relapse there is an increased prevalence of osteoporosis [68]. The adverse event hazard ratio is increased by approximately 3% for every 1000 mg increase in the cumulative GC dose [69].

## Recommendation

- Consider prescribing a proton pump inhibitor for gastrointestinal protection in people at risk of gastrointestinal bleeding or dyspepsia.
- Consider prescribing bone-sparing treatment, such as bisphosphonates, to people who are taking high doses of GC.

### Are conventional second line steroid sparing therapies effective in GCA?

There have been three randomised, placebo-controlled trials investigating Methotrexate (MTX), between doses of 7.5–15 mg per week, as an adjunctive steroid-sparing agent [70–72] Despite methodological limitations of each individual study, a meta-analysis using pooled individual patient data from these trials demonstrated a reduced risk of first relapse (HR 0.65, 95% CI 0.44 to 0.98, *p* = 0.04) and second relapse (HR 0.49, 95% CI 0.27 to 0.89, *p* = 0.02), a higher probability of glucocorticoid-free remission for at least 24 weeks (HR 2.84, 95% CI 1.52 to 5.28, *p* < 0.001) and a lower cumulative glucocorticoid dose (0.84 g less glucocorticoid use after 48 weeks in patients treated with MTX versus placebo). There was no significant difference in side effects between the intervention and placebo groups. On further review of the included studies a low dose of MTX had been used, compared with current practice and the patient selection included those in whom steroid titration from low doses had failed on several occasions [73]. Dumont et al. [67] recently reported a significant reduction in the duration of glucocorticoid treatment in half of their cohort with glucocorticoid-dependent disease who had received a steroid sparing agent (mainly but not exclusively methotrexate) of 36 [15—115] versus 61 [14—212] months (*p* = 0.008).

Other conventionally used steroid sparing therapies such as Azathioprine [74] [De Silva 1986], Cyclophosphamide [75] and Mycophenolate [76] have not been shown to be superior to GC alone. Ciclosporin was been deemed ineffective [77]. Use of anti-tumour necrosis factor (TNF) alpha therapy in GCA was disappointing [78]. Leflunomide has been investigated in an open-label prospective single centre study of 76 patients and shown some benefit in controlling disease, reducing the use of GC [79].

## Recommendation

Second line therapy with MTX should be considered in patients with GCA in order to maintain disease control and achieve glucocorticoid reduction, but the effectiveness of MTX is modest.

### What is the evidence for antiplatelet therapy in GCA?

Aspirin is widely used to prevent ischaemic complications such as stroke and myocardial infarction in those with cardiovascular disease. The literature has conflicting reports of the protective use of aspirin in GCA, with some supporting its beneficial effects [80, 81] and others refuting it [82, 83]. Currently overwhelming clinical evidence of a reduction in ischaemic events is lacking [84]. The use of low-dose aspirin as an adjunctive treatment in GCA must acknowledge the recognised haemorrhagic risks associated with aspirin and concurrent GC use.

## Recommendation

Clinician discretion and patient preference on initiating aspirin therapy in GCA may be applied, unless patients have associated pre-existing comorbid conditions which justify its use.

### What is the evidence for use of biologics in GCA?

Tocilizumab (TCZ), a humanised monoclonal antibody to the IL-6 receptor, has been investigated in phase II [85] and phase III [64] RCTs. In the phase II study 30 patients with new-onset or relapsing disease were randomised to receive either weekly TCZ infusions or placebo, both with a tapering GC regimen. At week 12, 85% of the TCZ group had achieved clinical and biochemical remission as compared with only 40% of those in the placebo group (*p* = 0.03). At 1 year, 85% of the TCZ group had remained relapse-free versus a mere 20% of the placebo group (*p* = 0.001) Use of TCZ resulted in a significant reduction in steroid requirement [86].

The Giant Cell Arteritis Actemra (GiACTA) trial was a large phase III, multi-centre, double-blind, placebo-controlled study examining the efficacy of subcutaneously administered TCZ to induce and sustain remission to 12 months. GiACTA included newly diagnosed patients with GCA and those with refractory disease. Patients were enrolled and randomised to one of four arms; weekly or fortnightly TCZ (162 mg) combined with a 26-week prednisolone taper or; placebo plus a 26-week or 52-week prednisolone taper. At 12 months, those receiving TCZ were significantly more likely to have achieved sustained remission as compared with both the 26-week and 52-week steroid taper groups. This confirmed a significant steroid-sparing effect of TCZ (patients receiving TCZ had required half the cumulative glucocorticoid dose used in the placebo groups) [64].

The side effect profile of TCZ (Table 1) is well documented in the rheumatoid arthritis literature [40]. Within the older GCA population concern has been raised regarding the risk of concurrent diverticular disease, transient neutropenias, and elevations of triglycerides and deranged liver function tests [87].

**Table 1:** Side effect profile for Tocilizumab [41]

Frequency	Side effect
Common	• abdominal pain • conjunctivitis • cough • dizziness • dyslipidaemia • dyspnea • gastrointestinal disorders • headache • hypersensitivity • hypertension • increased risk of infection • leucopenia • neutropenia • oral ulcers • peripheral oedema • skin reactions • weight increases
Rare	• hypothyroidism • nephrolithiasis
Very rare	• infusion related reaction • interstitial lung disease • pancytopenia • Stevens-Johnson syndrome

The cost, side effect profile and inability to serially monitor the serum CRP for evidence of relapse means that there is a reluctance to prescribe TCZ for new onset patients, despite the trial evidence. However, when patients are deemed refractory i.e. those people with GCA who never achieve remission, this is typically a more accepted use of TCZ. Likewise, for those patients with glucocorticoid-induced side effects or patients with significant pre-existing comorbidities such as psychiatric disturbances, pancreatitis, or uncontrolled diabetes or hypertension, tocilizumab could be effective as a first line treatment [21, 59].

The 2-year open-label extension study from GiACTA analysed 215 of the original 251 randomised patients. Participants in clinical remission received no further treatment and those not in clinical remission received weekly TCZ (162 mg) and/or GC and/or MTX. Higher proportions of those originally assigned to weekly TCZ were treatment-free compared with those originally assigned to placebo. Importantly, the cumulative dose of GC was substantially lower (almost half) in patients assigned to weekly TCZ (2604 mg) versus patients given either 26 weeks of GC plus placebo (5006 mg) or 52 weeks of GC plus placebo (5322.5 mg). Interestingly, patients assigned to placebo plus only 26 weeks of GC required almost as much GC therapy as those assigned to 52 weeks of GC plus placebo, reflecting the poor control of disease when using 26 weeks of GC monotherapy [86].

### Recommendation

Use of TCZ should be considered for patients with a confirmed diagnosis of GCA (based on imaging or TAB) who have either refractory disease, or those with co-morbid disease that could be significantly exacerbated by GC use.

## Conclusions

There have been significant advances occurring to routine clinical GCA care. Guideline bodies from Europe have updated their recommendations to rheumatologists [20, 59, 88] and neurologists need to be up to date to ensure optimal evidence-based practice for their patients.

This EHF guidance serves to inform neurologists who may be consulted regarding a patient with a new onset headache over the age of 50 years old, and who indeed may manage patients with GCA long-term. With advances in imaging and treatment it is no longer recommended to diagnose GCA on clinical grounds alone. A confirmatory investigational test such as CDUS or TAB is required, because the disease course of an individual currently cannot be readily predicted at diagnosis and escalation in therapy may be needed, but eligibility for escalation therapy is likely to require prior evidence of a definitive diagnosis.

An appreciation of the growing evidence regarding glucocorticoid morbidity is required in this population. Randomized clinical trials have shown that traditional steroid sparing agents perform only modestly; by contrast, biologic agents such as TCZ have clinically relevant efficacy in GCA. As other future targeted therapies are on the horizon (Table 2), and as our understanding of the disease entity becomes more informed this guidance will require timely updating.

**Table 2:** Drugs currently being investigated for treatment of GCA

Drug name	Mechanism of action
Abatacept	Humanized fusion protein that modifies co-stimulation in antigen presentation, inhibiting T-cell activity
Anakinra	Monoclonal antibody to the IL-1β receptor
Baricitinib	Synthetic DMARD, which targets the intracellular pro-inflammatory Janus kinase (JAK) family of enzymes
Gevokizumab	Recombinant monoclonal antibody to IL-1β, a pro-inflammatory cytokine
Rituximab	Chimeric monoclonal antibody against the protein CD20, which is primarily found on the surface of immune system B cells

## Supplementary information

**Additional file 1: Movie S1.** Whole body FDG-PET movie illustrating increased metabolic activity in the aorta (most pronounced in the descending part) as well as some of its main branches (including both vertebral arteries, more pronounced on the left).

**Additional file 2: Case Vignette 1.** the overlap of LV-GCA, cranial GCA and PMR. Case Vignette 2: Utility of FDG PET imaging to diagnose GCA.

## Data Availability

All data generated or analysed during this study are included in this published article and its supplementary information files.

## Abbreviations

CDUS: Colour Duplex Ultrasonography
CRP: C-reactive protein
CT: Computed tomography
CTA: Computed tomography angiography
DMARDs: Disease modifying anti-rheumatic drugs
EHF: European Headache Federation
ESR: Erythrocyte sedimentation rate
FDG: Fluorodeoxyglucose
GiACTA: The Giant Cell Arteritis Actemra Trial
GCA: Giant cell arteritis
IHS: International Headache Society
MRI: Magnetic resonance imaging
MXT: Methotrexate
PET: Positron emission tomography
PMR: Polymyalgia rheumatica
RCT: Randomised controlled trial
TAB: Temporal artery biopsy
TCZ: Tocilizumab
TNF: Tumour necrosis factor
