# Supplementary material for: European Headache Federation recommendations for neurologists managing giant cell arteritis
Source: J Headache Pain. 2020 Mar 17;21(1):28. doi: 10.1186/s10194-020-01093-7 (PMC7079499; doi:10.1186/s10194-020-01093-7)
Supplement: Supplementary file 2 — Additional file 2: Case Vignette 1. the overlap of LV-GCA, cranial GCA and PMR. Case Vignette 2: Utility of FDG PET imaging to diagnose GCA. [file 10194_2020_1093_MOESM2_ESM.docx]

**Case Vignette 1: the overlap of LV-GCA, cranial GCA and PMR.**

A 72-year-old Caucasian woman presents with bilateral frontotemporal as well as occipital headache for 2 weeks, worse at night time. There are no visual complaints nor jaw claudication. Systems review is positive for weight loss. She is on a low dose of methylprednisolone (4 mg/day), started 3 months earlier at a dose of 8 mg/day based on a working diagnosis of polymyalgia rheumatica as she complained of mainly nocturnal neck and shoulder muscle aching with morning stiffness but with an ESR consistently below 30 mm/hr.

On physical examination, there is no tenderness of the scalp over the temporal areas, no thickening of the temporal arteries and pulsations are normal. Ophthalmological examination revealed normal optic discs. There are no carotid or subclavian bruits, and the blood pressure is normal and equal in both arms. The remainder of the examination is unremarkable. However, blood testing reveals significantly increased inflammatory parameters (including ESR of 98 mm/hr and C-reactive protein level of 116,5 mg/L) as well as elevated transaminases.

A whole body FDG PET-CT revealed increased metabolic activity in both temporal arteries (more pronounced on the left, figure 2), in the aorta (most pronounced in the descending part, figure 3 and movie 1) as well as in some of its main branches (including both vertebral arteries, more pronounced on the left); there is no evidence of malignancy. Gadolinium-enhanced MRI of the brain was unremarkable. No temporal artery biopsy was performed. Appropriate treatment for GCA was initiated.

**Case Vignette 2: Utility of FDG PET imaging to diagnose GCA.**

A 56-year old male presented with a 30 year history of an unspecified immune mediated inflammatory disorder that has required high dose glucocorticoids for the preceding one year. He reported episodic fever at night, shoulder pain and active psoriasis. He had raised inflammatory parameters (ESR of 86 mm/hr, 441x10^3^ platelets and a CRP of 186 mg/L). An FDG PET scan was performed (figure 4) after which the diagnosis of GCA is made. He was initially treated with oral prednisone 40 mg with a good clinical response. More than 6 months later, methotrexate was added. A complete remission was achieved almost 2 years after diagnosis.
